# Simplest Way to Establish COVID-19 Quarantine Observation Wards Within 24 Hours

**DOI:** 10.1177/1010539520947874

**Published:** 2020-08-14

**Authors:** Wen-Nan Huang, Mao-Song Zhuang, Tsun-Jen Cheng, Shih-Huai Hsiao

**Affiliations:** 1 Kaohsiung Medical University Hospital, Kaohsiung Medical University, Kaohsiung, Taiwan; 2 Kaohsiung Municipal Ta-Tung Hospital, Kaohsiung Medical University, Kaohsiung, Taiwan; 3 Kaohsiung Medical University, Kaohsiung, Taiwan

**Keywords:** COVID-19, nosocomial transmission, negative pressure, airborne infection isolation rooms, health care–associated infections

## Abstract

Reducing nosocomial transmission within health care facilities is important, but the number of negative-pressure airborne infection isolation rooms for SARS-CoV-2 (severe acute respiratory syndrome coronavirus 2) is limited. It is a daunting challenge to cope with a surge of suspected infectious patients in hospitals. We installed air exhaust fans on the windows to change the pressure direction within the wards rapidly. The best location for the fans was 90 cm from the floor and 90 cm from the edge of bed whether the indoor air conditioners were on or off. The noise level should be <60 dB(A) as per government regulations. General wards can be transformed into makeshift negative-pressure rooms easily and effectively within 24 hours, which is really the simple, fast, and effective way for the transformation being applied.

Most Taiwan hospitals have used negative-pressure airborne infection isolation rooms (AIIRs) to house patients with suspected or confirmed airborne transmissible infections since the 2003 severe acute respiratory syndrome epidemic,^1^ but the number of AIIRs is limited. With the ongoing novel coronavirus disease 2019 (COVID-19) pandemic, the number of individuals who need to be tested for COVID-19 has been increasing rapidly, while there are no regulations stipulating capacity requirements for hospitals, especially tertiary care (medical center) institutions. To cope with a surge of patients in hospitals who are suspected of the infection is challenging.^2^ In addition, suspected cases should not be permitted to wait alongside other patients seeking medical care for other complaint diseases. They should be placed in a separated, fully ventilated room and approximately 2 m away from other patients with convenient access to respiratory hygiene supplies.^3^

Kaohsiung Medical University Hospital (KMUH) started to prepare for COVID-19 on January 21, 2020, and infection control measures were tightened to limit nosocomial transmission and health care–associated infection within health care facilities.

With an average of 9,838 people per square kilometer at Kaohsiung city, the population density of Taiwan ranks second highest worldwide, with 80% of its population concentrated in six municipalities. Moreover, Taiwan is one of only five countries and regions in the world that had not yet announced full suspension of schools and classes or required citizens to stay at home by March 31, 2020. Consequently, when it comes to the reception and treatment of suspected cases with SARS-COV-2 (severe acute respiratory syndrome coronavirus 2; COVID-19), it is not feasible to build mobile cabin hospitals with makeshift isolation wards or walk-through negative-pressure booths by expropriating hotels, schools, parks, or spacious buildings within cities, walk-through,^4^ or drive-through experience.^5,6^ How to designate a specific hospital to diagnose and preempt quarantine patients with suspected COVID-19 and acute respiratory symptoms as a COVID-19 hospital is an essential challenge. This COVID-19 safety hospital will also operate a screening center during the COVID-19 epidemic. At the screening centers, prompt screening and specimen collection are important, but the safety of medical staffs and testees is the top priority.^6^ Cross-contamination should also be minimized during the specimen collection process^7^ and while waiting for the test result.

It is suggested that we either make use of existing wards in hospitals, as their utilization rate has fallen due to the COVID-19 outbreak, or build outdoor parking lots or transform them into makeshift quarantine observation wards. This would be the only way to respond to the influx of suspected cases into hospitals and to contain further infection as patients have to stay in negative-pressure booths while waiting for test results.

KMUH, a medical university affiliated hospital, has twice obtained Joint Commission International accreditation. It is a large-scale hospital with 1,710 beds, which provides outpatient services for 1.98 million person-times annually, and its annual length of stay is 546,500 days. In response to a surge in suspected patients after the outbreak of COVID-19, the hospital has been using general wards as quarantine observation wards for suspected patients while they wait for the test results of second-time screening.

The partitions in the wards are closely connected to the floor slabs, and windows stay tightly closed. Each ward is equipped with small and independent air conditioners (12 m^3^, including 2 m^3^ air supplement system for precooling external fresh air) for indoor circulation, airflow speed control, and cooling. Air exhaust fans are installed on the windows to change the pressure direction within the wards rapidly. Mist generators with 0.58 CC mist/minute and a maximum jet height of 50 cm based on ultrasonic wave oscillation are used to measure and optimize the direction of airflow so as to identify the best location for exhaust fan installation. After testing, the results are as follows:

1. Installation height of exhaust fans (Table 1)
  1.1. Installed at a height of over 90 cm above the ground, the exhaust fan is unable to effectively expel the mist over a standard hospital bed and the mist scatters in a nondirectional way, which indicates that the droplets from the patients are likely to scatter and drift indoors, causing higher risk of infection for the health care workers attending patients.
  1.2. Installation height of exhaust fans has no effect on the airflow from corridor into the ward. As the mist from the nursing station to corridor and into the ward keeps flowing in the correct direction, it means contaminated air in the ward is under effective control without outflow.
  1.3. Regardless of the installation height of exhaust fans or the distance between the fan and the bed, indoor conditioners being on or off and air volume have no effect on mist flow direction. Therefore, when the patients or family members turn off the indoor AC, outflow of air from the contaminated zones to the corridors and nursing stations is not an issue for concern.
2. Distance between the bed and the exhaust fan
  2.1. Exhaust fans installed 60 cm from the bed edge work best to expel mist. However, when the patient is in bed, the level of noise from the fan (63 dB(A)) exceeds the legal limit.
  2.2. If the distance is 90 cm, the noise level will decrease to 58.2 db. On one hand, we should not move the bed so as not to compromise air-exhaust performance. On the other hand, noise level should be under control so that it will not cause discomfort or anxiety to patients.^8^ Therefore, a designated area for the bed should be marked on the floor.

**Table 1.** Testing Results in Makeshift Quarantine Observation Wards After Installation of Exhaust Fans.

Ward	Total indoor volume (m^2^)	Technical specifications of exhaust fans	Installation location		Indoor AC		Airflow direction in corridor to ward^a^	Airflow condition at bedside		Level of noise (dBA)	
			Height above ground (cm)	Minimum distance to bed edge (cm)	On/off	Air volume (CMM)		To fans	From bedside to fans	Background	Patients lying on the bed^b^
6C	47.2	Model LH-148/3 blades/dimension: diameter 30 cm/air volume unknown/level of noise: unknown/56 W	180	120	On	17	+++	—	—	51.2	55.8
6C	47.2		180	120	Off	0	+++	—	—	51.2	54.6
6C	47.2		90	120	On	17	+++	+	—	51.2	56.4
6C	47.2		90	120	Off	0	+++	+	—	51.2	55.3
6C	47.2		90	100	On	17	+++	+	+	51.2	56.4
6C	47.2		90	90	On	17	+++	+	+	51.2	58.2*
6C	47.2		90	70	On	17	+++	++	+	51.2	61.5
6C	47.2		90	60	On	17	+++	+++	++	51.2	63.2
6C	55.4	Model JFB-14H/3 blades/dimension: diameter 55 cm/air volume: 55 CMM/level of noise:52dB(A)/35 W	180	120	On	17	+++	—	—	53.5	56.3
6C	55.4		180	120	Off	0	+++	—	—	53.5	56.4
6C	55.4		90	120	On	17	+++	—	+	53.5	57
6C	55.4		90	120	Off	0	+++	—	+	53.5	56.2
6C	55.4		90	110	On	17	+++	+	+	53.5	55.1
6C	55.4		90	100	On	17	+++	++	++	53.5	55.4
6C	55.4		90	90	On	17	+++	+++	++	53.5	54.7

a +++, visible smooth flow; ++, visible little smooth flow; +, visible little smooth flow but slow.

b dB(A)-Leq24 is measured by a decibel meter, which is certificated by the Office of Occupational Safety and Health Administration, Taiwan.

There were a lot of empirical studies discussing and demonstrating how to build AIIRs,^9-11^ but to build a new AIIR is time consuming. The above-mentioned measures have been applied in the transformation of general wards into negative-pressure isolation rooms in Taiwan. Despite COVID-19 virus, exhaust fans are available on the market as they are not affected by international logistics and there is no need of customization or waiting for resumption of production lines. We found that a general inpatient ward area with 30 beds can easily, quickly, and effectively be transformed into makeshift negative-pressure rooms within 24 hours.

## References

[1] Hsu YC Chen YL Wei HN Yang YW Chen YH Risk and outbreak communication: lessons from Taiwan’s experiences in the Post-SARS era. Health Secur. 2017;15:165-169. 28418746 10.1089/hs.2016.0111 PMC5404243

[2] Miller SL Clements N Elliott SA Subhash SS Eagan A Radonovich LJ Implementing a negative-pressure isolation ward for a surge in airborne infectious patients. Am J Infect Control. 2017;45:652-659. 28330710 10.1016/j.ajic.2017.01.029 PMC7115276

[3] Harapan H Itoh N Yufika A, et al Coronavirus disease 2019 (COVID-19): a literature review. J Infect Public Health. 2020;13:667-673. 32340833 10.1016/j.jiph.2020.03.019 PMC7142680

[4] Kim SI Lee JY Walk-through screening center for COVID-19: an accessible and efficient screening system in a pandemic situation. J Korean Med Sci. 2020;35:e154. 10.3346/jkms.2020.35.e154 PMC7167399 32301300

[5] Weiss EA Ngo J Gilbert GH Quinn JV Drive-through medicine: a novel proposal for rapid evaluation of patients during an influenza pandemic. Ann Emerg Med. 2010;55:268-273. 20079956 10.1016/j.annemergmed.2009.11.025

[6] Lee J COVID-19 screening center: how to balance between the speed and safety? J Korean Med Sci. 2020;35:e157. 10.3346/jkms.2020.35.e157 PMC7167407 32301302

[7] World Health Organization. Laboratory testing for coronavirus disease 2019 (COVID-19) in suspected human cases. Accessed July 24, 2020 https://apps.who.int/iris/bitstream/handle/10665/331329/WHO-COVID-19-laboratory-2020.4-eng.pdf?sequence=1&isAllowed=y

[8] Wright BA Peters ER Ettinger U Kuipers E Kumari V Moderators of noise-induced cognitive change in healthy adults. Noise Health. 2016;18:117-132. 27157685 10.4103/1463-1741.181995 PMC4918666

[9] Cheong K Phua S Development of ventilation design strategy for effective removal of pollutant in the isolation room of a hospital. Build Environ. 2006;41:1161-1170.

[10] Adams NJ Johnson DL Lynch RA The effect of pressure differential and care provider movement on airborne infectious isolation room containment effectiveness. Am J Infect Control. 2011;39:91-97. 20864218 10.1016/j.ajic.2010.05.025

[11] Tsai YH Wan GH Wu YK Tsao KC Airborne severe acute respiratory syndrome coronavirus concentrations in a negative-pressure isolation room. Infect Control Hosp Epidemiol. 2006;27:523-525. 16671039 10.1086/504357

